# Anomalously high geothermal flux near the South Pole

**DOI:** 10.1038/s41598-018-35182-0

**Published:** 2018-11-14

**Authors:** T. A. Jordan, C. Martin, F. Ferraccioli, K. Matsuoka, H. Corr, R. Forsberg, A. Olesen, M. Siegert

**Affiliations:** 1 British Antarctic Survey, High Cross, Madingley Road, Cambridge, CB3 0ET UK; 2 Norwegian Polar Institute, Tromsø, Norway; 3 National Space Institute, Technical University of Denmark, Lyngby, Denmark; 4 Grantham Institute and Department of Earth Sciences and Engineering, Imperial College London, South Kensington, London, SW7 2AZ UK

## Abstract

Melting at the base of the Antarctic Ice Sheet influences ice dynamics and our ability to recover ancient climatic records from deep ice cores. Basal melt rates are affected by geothermal flux, one of the least constrained properties of the Antarctic continent. Estimates of Antarctic geothermal flux are typically regional in nature, derived from geological, magnetic or seismic data, or from sparse point measurements at ice core sites. We analyse ice-penetrating radar data upstream of South Pole revealing a ~100 km long and 50 km wide area where internal ice sheet layers converge with the bed. Ice sheet modelling shows that this englacial layer configuration requires basal melting of up to 6 ± 1 mm a^−1^ and a geothermal flux of 120 ± 20 mW m^−2^, more than double the values expected for this cratonic sector of East Antarctica. We suggest high heat producing Precambrian basement rocks and hydrothermal circulation along a major fault system cause this anomaly. We conclude that local geothermal flux anomalies could be more widespread in East Antarctica. Assessing their influence on subglacial hydrology and ice sheet dynamics requires new detailed geophysical observations, especially in candidate areas for deep ice core drilling and at the onset of major ice streams.

## Introduction

The East Antarctic Ice Sheet drains to the coast through conduits of fast flowing ice, known as ice streams, from an interior dominated by slow moving ice of the high, cold, polar-desert. The underlying East Antarctic continent is generally considered to be an ancient craton, last affected by a major tectono-thermal event ~500 Ma^1^, although many provinces are much older^2,3^. More recent (300-100 Ma) tectonic rifts dissect East Antarctica but appear not to have significantly altered the underlying Precambrian lithosphere^4^. Geophysical techniques provide regionally averaged estimates of geothermal flux and yield low values (50–60 mW m^−2^)^5–7^ for the composite East Antarctic craton, in line with global averages for cratonic heat flux^8^. Such regional estimates form the basis for coupled climate-ice sheet models suggesting variations in geothermal flux have limited impact on ice sheet stability^9^. More recent modelling studies indicate, however, that locally elevated geothermal flux can affect ice flow patterns in the interior of East Antarctica^10^. Although predicted by some geological studies of coastal regions^11^ direct evidence for the required local geothermal anomalies beneath the East Antarctic Ice Sheet was lacking.

**Figure 1:**
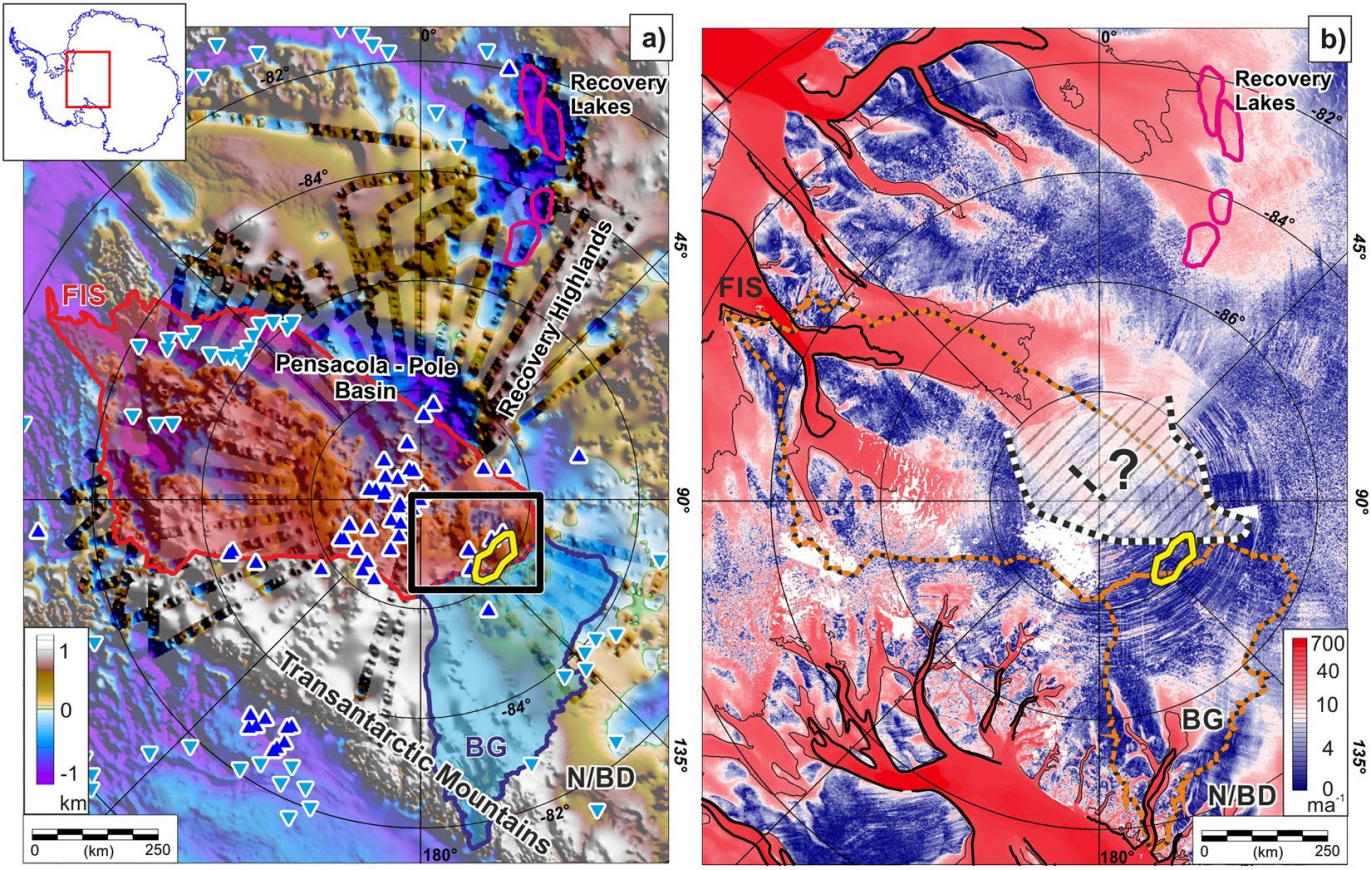
Regional setting. (**a**) Subglacial topography from new PolarGAP survey (strong colours), ICEGRAV^56,57^ and BEDMAP2^58^. Yellow outline marks region of enhanced basal melting. Pink outlines are Recovery Lake shorelines^26^, dark and pale blue triangles mark ‘static’ radar-detected and ‘dynamic’ satellite-detected subglacial lakes, respectively^17^. Foundation Ice Stream (FIS) and Beardmore Glacier (BG) catchments marked in red and blue respectively. N/BD indicates Nimrod/Byrd Glacier catchments. Black box locates Fig. 2. (**b**) Present ice velocity map^19^. Thick and thin black contours show 100 and 25 m a^−1^ flow velocities respectively. Orange lines mark FIS and BG catchments. Note region of former enhanced flow to FIS^13^ (grey hash) extending ~250 km further inland and black dashed line close to South Pole marking a relic shear margin^16^.

**Figure 2:**
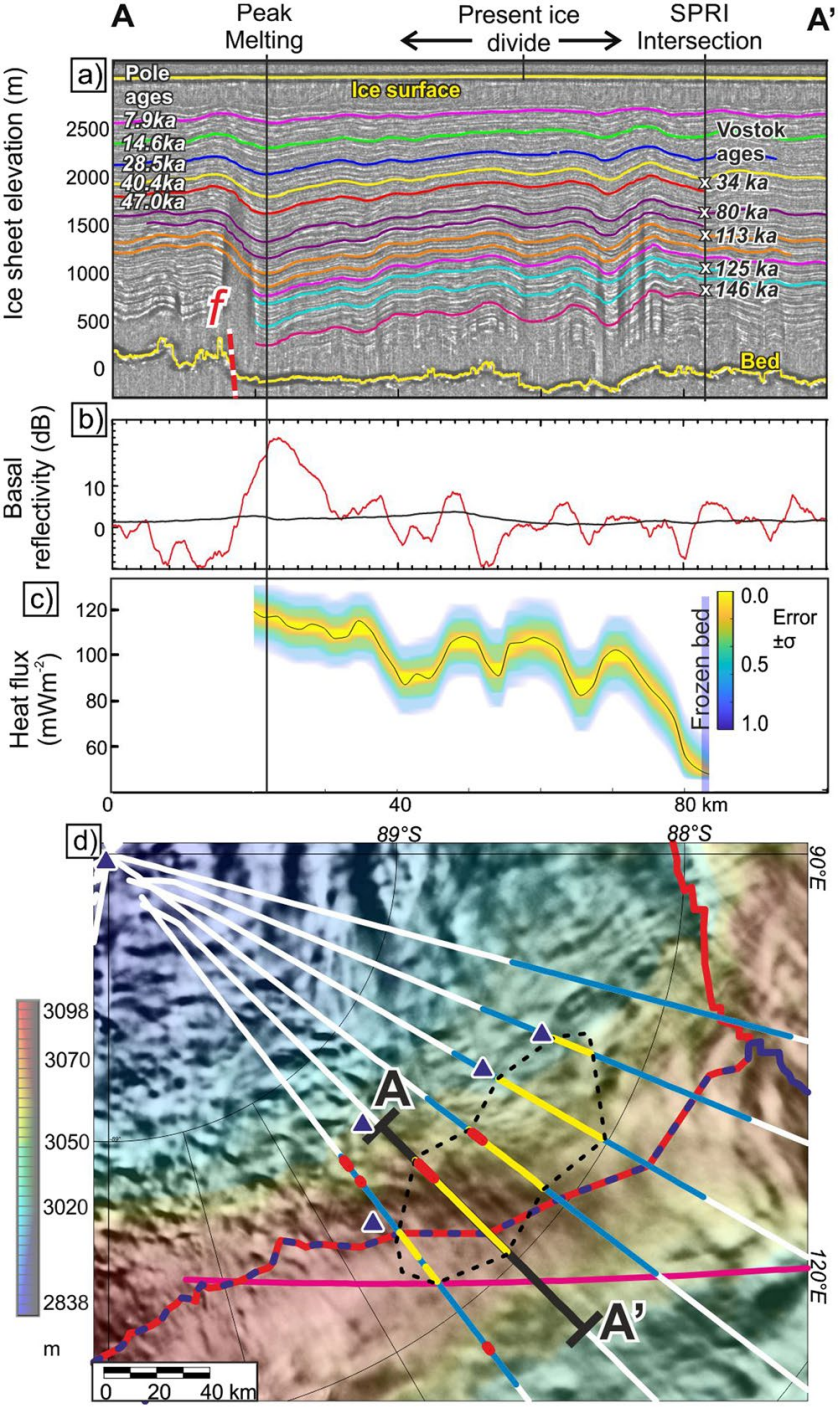
Model profile across area of enhanced basal melting. (**a**) Depth converted radargram showing internal layer drawdown (See Supplementary Figure 1b for background data). Coloured lines show manually traced layers used to calculate melt rate. Ages from South Pole and Lake Vostok used to construct initial depth age model at the intersection with older aerogeophysical survey line (SPRI)^54^. Red dashed line (*f*) marks inferred fault location. Black arrows indicate surface ice flow direction. (**b**) Bed brightness. Red line shows corrected brightness with a 3 km mean filter. Black line shows regional (60 km mean) reflectivity. Areas >10 dB above the regional level indicate subglacial water. (**c**) Modelled geothermal heat flux. The +/−1 sigma error bounds were calculated based on the distribution of the results of 1000 runs of our Monte Carlo analysis. In the area of frozen bed (blue bar) basal melting is modelled to be zero, and only a maximum estimate of heat flux can be made. (**d**) Our new surface elevation grid (Supplementary Material Section 1.3 and 4) overlain with MOA imagery showing PolarGAP flights (white). Black line locates profile A-A’. Blue sections shown in Fig. S1. Yellow lines mark observed layer draw down. Red areas indicate bright bed >10 dB above regional values. Pink line locates SPRI profile. Dashed red-blue line marks topographic ice-flow divide between Foundation (upper left) and Beardmore Glacier (lower right) catchments. Blue triangles are small (<5 km) subglacial lakes imaged in older surveys^17^.

Here we make use of englacial layers observed with ice-penetrating radar as an alternative way to assess geothermal heat flux variability beneath the East Antarctic Ice Sheet in greater detail than possible from continental scale geophysical datasets. Specifically, we examined new radar data collected as part of the ESA PolarGAP campaign over the South Pole region^12^ (Fig. 1a and Supplementary Material 1). This data provides a direct measurement of ice thickness, revealing major subglacial basins extending from the Weddell Sea over 750 km into the interior of East Antarctica (Fig. 1a). This data also images internal layers within the ice sheet, attributed to isochronous deposition of snow layers with specific chemical characteristics. The pattern of such layers within the ice sheet reflects the interplay between accumulation, basal melting and ice sheet deformation^13^. The survey area extends radially ~700 km from South Pole and ice flow typically dominates internal layer geometry. However, upstream of South Pole, close to the ice divide between the Foundation and Beardmore Glacier catchments, ice flow is slow (Fig. 1b) allowing examination of basal processes using ice sheet internal layering (Fig. 2). This is an area of particular interest as models suggest it may contain some of the planet’s oldest ice, preserving records of important climatic transitions^14^. These models of ancient ice distribution are influenced by borehole temperature measurements indicating low geothermal flux and frozen bed at South Pole^15,16^. In contrast, numerous subglacial lakes in this region^17^ suggest enhanced basal melting. Some studies attribute basal melting to former enhanced ice flow speeds^16^ rather than elevated geothermal flux, supported by evidence of so-called organised flow^13^ and a buried shear margin^16^ close to South Pole (Fig. 1b).

## Results

### Present-day basal conditions

**Figure 3:**
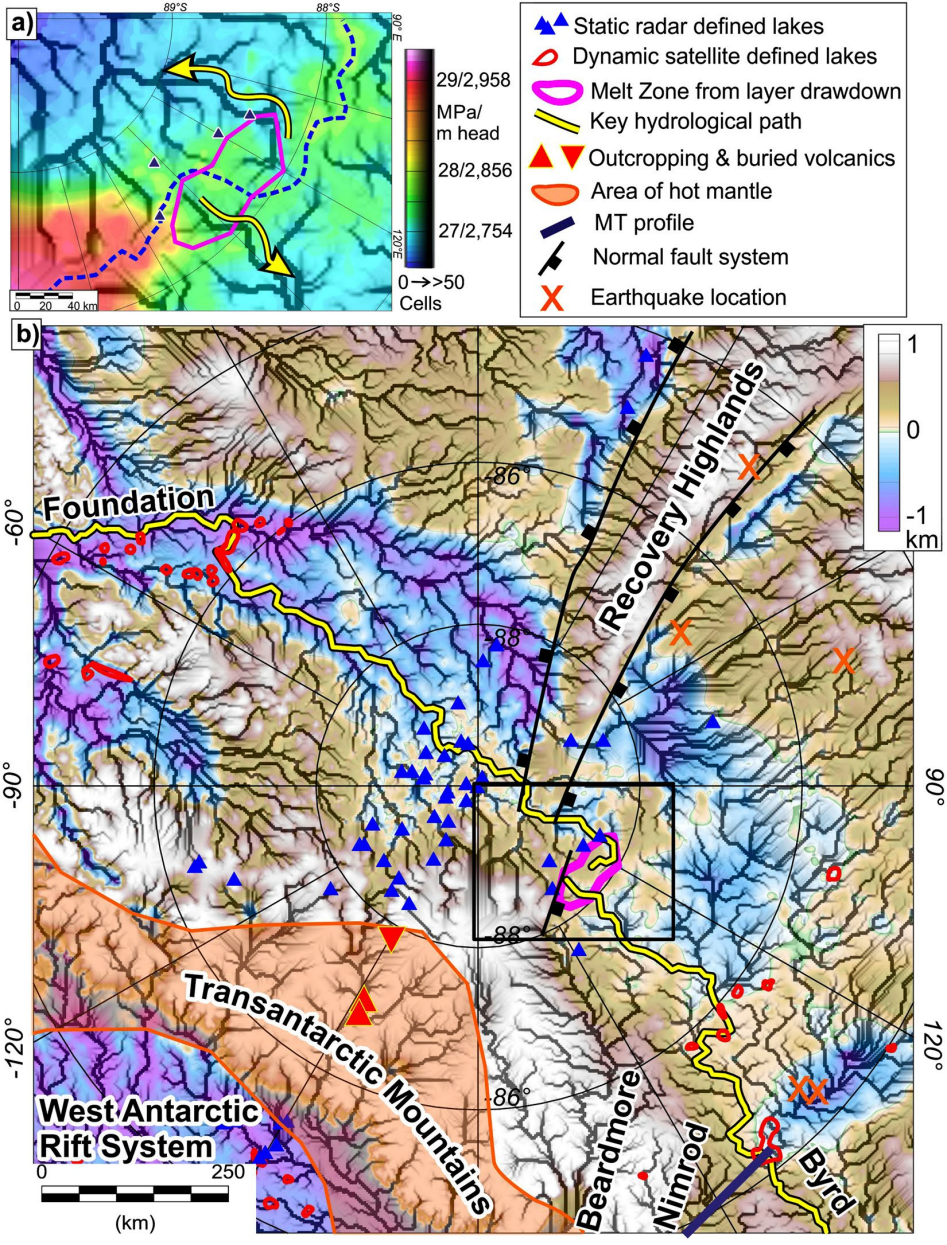
Hydrological model and geological setting. (**a**) Detail of present day hydrological potential and results of flow accumulation model^31,32^. Grey shading indicates cumulative number of upstream cells, and hence effective drainage pathways. Note we chose an arbitrary threshold of 50 upstream cells to define an established hydrological pathway. Dashed blue line shows hydrological divide. (**b**) Regional hydrological pathways on new sub-ice topography, with tectonic and geological features overlaid. Note melt water modelled to flow via present dynamic lakes further down the catchments.

Close to the ice divide upstream of South Pole multiple radar profiles show stratigraphic englacial layers throughout the ice sheet that are drawn down towards the bed (Figs 2 and S1). Figure 2 shows the least topographically complex of the upstream profiles (Fig. S1). We therefore use this profile to illustrate and model the observed layer drawdown of more than 400 m that occurs over a horizontal distance of ~50 km (Fig. 2a). Layer drawdown resulting from out of plane ice flow^18^ is unlikely to be an explanation for the layer geometry observed here as the ice velocity^19^ is slow (~1.5 m a^−1^) and significant upstream topographic variations, which can modify along flow layer geometry^20^, are not observed. We therefore attribute the observed loss of ice to enhanced basal melting^21–23^. The greatest level of drawdown overlies an area where bed reflectivity is >10 dB higher than the regional mean (Fig. 2b). We interpret this area of high reflectivity, relative to the rest of the profile, as an indicator of discontinuous basal water, following previous interpretations of enhanced basal reflectivity in other regions^24^ (Fig. 2b and Supplementary materials 1 and 2). If a major subglacial lake was present the ice sheet bed would be in hydrostatic equilibrium with the surface slope^25^. This is only true for a region ~2 km long in the area of brightest bed, indicating a more extensive subglacial lake is not present. The internal layer draw-down, supported by the highly-reflective bed, points to active and significant basal melting in this region. The location of the melting, almost at the ice divide and near the head of the calculated hydrological catchment (Fig. 3a), supports a local origin for the basal water, rather than from inflow from a more distant source.

To quantify the spatial extent of the melting we consider six radar profiles close to the ice divide (Fig. S1). As topography is more complex on several of these profiles the pattern of englacial layers normalised for ice thickness is also considered. The central profiles show regions of clear layer drawdown 35–50 km long, which in places correlate with highly reflective bed (Fig. 2d). Adjacent lines show more limited regions of layer drawdown, indicating an overall area of layer drawdown ~100 km long and ~50 km wide, approximately equivalent to the area of the largest of the Recovery Lakes^26^. A number of small (<5 km long) subglacial lakes identified in older reconnaissance radar data also point to basal melting in this area^17^ (Fig. 2d). However, we are not able to specifically re-identify these lakes, which we attribute to the poor navigational information available in the older survey data.

### Melt rates and geothermal flux

By modelling the depth age profile of the internal layers (see Methods and Supplementary Material 2 and 3) we estimate peak melt rates of 6 ± 1 mm a^−1^ (Fig. S5). This melt rate is at least double that predicted by previous regional models^27^ and equivalent to rates associated with the upstream parts of the Whillans Ice Stream in West Antarctica^28^. The associated average estimate of surface accumulation for the last thousand years is ~47 mm a^−1^ (Fig. S6). The lateral variations in surface accumulation are comparable in scale with the patterns seen elsewhere in Antarctica^29^. The geothermal flux required to generate the largest observed melt rates is 120 ± 20 mW m^−2^ (Fig. 2c), with background values where no melting is predicted of 48 mW m^−2^ or less. Basal shearing as ice flows over subglacial topography can be an important contributor to the basal heat budget and melting^16,30^. However, the low surface ice velocity in this region (~1.5 m a^−1^) implies extremely low ice velocity at the ice-bedrock interface (≪1.5 m a^−1^), and we can therefore bound the heating due to basal shearing^30^ to be smaller than 1 mW m^−2^. In our analysis, we neglect the small contribution of basal shearing to the heat budget and hence assume that the observed basal melting is due to geothermal heat flux. The estimated peak geothermal flux values are twice those indicated by previous continental-scale estimates derived from passive seismic and magnetic methods^5–7^.

### Hydrological routing

Enhanced basal melting due to anomalously high geothermal flux has likely been a constant factor on geological (>1 Ma) time scales. The impact of the imaged enhanced subglacial melting on East Antarctic Ice Sheet dynamics will depend on the routing of melt water. Basal and surface topography are the critical controls on subglacial water routing, but were poorly constrained in this region. Our new bed and ice surface topography provide improved boundary conditions (Figs 1a and 2d) allowing water routing to be more accurately calculated using the hydrological potential (Fig. 3)^31,32^ (Supplementary Material 1 and 4). Uncertainties in topography and hence hydrological potential remain and are difficult to quantify, but our model is the best possible with available data. Our hydrological model shows that today drainage of water generated in the area of layer drawdown splits approximately evenly between the Foundation and the Byrd Glacier catchments (Fig. 3a). Flow paths indicate that melt water flows into inferred dynamic lakes^33^ in downstream areas both sides of the main ice divide (Fig. 3b). Hence, given the influence that basal water typically exerts on ice sheet dynamics^34,35^, we propose that the geothermally enhanced basal melting at the South Pole ice divide is likely to have affected present and past East Antarctic Ice Sheet flow across a number of glacial catchments. Such a long-term water source may have helped provide the necessary boundary conditions for the enhanced and organised ice flow observed in the South Pole region^13,16^.

## Discussion

The cratonic nature of the interior of East Antarctica means elevated geothermal heat flux is an unexpected result^8^. A number of geological factors could cause the observed elevated geothermal flux and basal melting. The adjacent West Antarctic region is predicted by many studies to be a region of elevated geothermal flux^5,7,36,37^, linked to the existence of a major Cretaceous to Cenozoic rift system^38^. Notably, recent passive seismic studies have revealed warm West Antarctic mantle impinging beneath the Transantarctic Mountains towards the South Pole region^39^ (Fig. 3b). However, hot mantle as the direct source for the observed geothermal anomaly is unlikely given the localised area of identified ice sheet melting and low background geothermal flux values we found. Cenozoic magmatism associated with the warm mantle could generate a localised geothermal anomaly via intrusions or volcanism. Outcrops^40^ and erratics^41^ show Cenozoic (17–20.6 Ma) volcanics are present in neighbouring sectors of the Transantarctic Mountains (Fig. 3b). Aeromagnetic data suggests the sub-ice outcrops, which are the likely source of the volcanic erratics, are within the region of the warm mantle intrusion, but do not extend further towards South Pole^41,42^. This observation, in conjunction with the magnetotelluric^43^ and seismic imaging of thick cold lithosphere^39^ towards the area of elevated geothermal heat flux we identify means we do not favour a West Antarctic, or associated Cenozoic volcanic source for the observed geothermal feature.

Although cratons generally show low geothermal flux^8^ the Central Australian Heat Flow Province^44^ provides an example of a cratonic region with anomalously high geothermal flux. High heat flux values within a craton are due to radiogenic granitoids in the upper crust, which give rise to local geothermal anomalies. In the Central Australian Heat Flow Province, Meso and Paleoproterozoic (ca 1800-1600 Ma) granitoids cause locally high geothermal heat flux and likely also underlie parts of the East Antarctic conjugate margin^11^. In other areas of East Antarctica Cambrian granites^11^ have been recognised which could create local geothermal anomalies of up to 120 mW m^−2^, and radiogenic Jurassic granites in the Ellsworth-Whitmore Mountains microcontinent^45^ are modelled to give geothermal anomalies of up to 95 mW m^−2^. Geothermal anomalies in the East Antarctic interior generated by radiogenic source rocks depend on such rocks being present. Provenance studies from the catchments of the Byrd and Nimrod glaciers (Fig. 3b) indicate the basement is composed largely of Proterozoic (1.2–2.0 Ga) granitoids^46^. Low heat production is recorded in 16 out of the 17 basement samples^47^, but one 1850 Ma sample stands out as being highly radiogenic, giving a predicted heat flux of 83.6 mW m^−2^. The presence of this sample indicates that although sparse, it is reasonable to infer that high heat producing granitoids exist in the South Pole region of East Antarctica. Additionally, it is important to consider that radiogenic intrusions could be buried and hence be more widespread than erratics alone appear to indicate.

The peak geothermal flux predicted from erratics in the Transantarctic Mountains^47^ is 83.6 mW m^−2^, and requires a heat production of ~7.5 μW m^−3^. This heat flux is ~35 mW m^−2^ above background, but significantly less than our modelled value of 120 mW m^−2^. Studies in other parts of Antarctica^45^ have shown that heat flux 30 mW m^−2^ above the regional background requires a highly radiogenic granite 8 km thick with a mean heat production of 5.35 μW m^−3^. Models^11^ indicate that a thermal anomaly of ~120 mW m^−2^ requires heat production of ~50 μW m^−3^, almost an order of magnitude above anything measured in the Transantarctic Mountains erratics^47^. An additional mechanism is therefore likely required to explain the observed geothermal anomaly. Hydrothermal circulation, in particular where faults provide a rapid conduit to the surface for water heated at depth, is a mechanism that can act to enhance local geothermal flux. Studies in the US Basin and Range province^48^, along the Têt fault system in the French Pyrenees^49^, and in the Rhine Graben^50^ demonstrate that thermal anomalies resulting from fluid circulation within the brittle upper crust are significant and can more than double local geothermal flux. The geothermal anomaly we identify lies at the foot of a ~450 m escarpment at the boundary between a broad flat plain and a topographic highland (Fig. 2a). Although radar data alone does not uniquely define faults, this bedrock configuration is consistent with the presence of a partly eroded fault scarp adjacent to a half graben, as seen for example in mainland Greece^51^. We therefore propose that hydrothermal circulation, influenced by a fault system, is a potential additional explanation for the high amplitude of the geothermal anomaly we model. Notably, the fault scarp we infer lies along strike from the Recovery Highlands (Fig. 3b). This uplifted fault-bounded block is located at an elevation of ~1000 m above a branch of the Permian to Cretaceous East Antarctic Rift System that dissects East Antarctica^4^. Recent earthquake focal solutions support the interpretation that this is a fault bounded mountain range and indicates that some of the faults systems flanking the range are still active^52^. Such active faulting is likely to help maintain high permeability along the fault system, facilitating continued geothermal circulation, thereby contributing to enhanced geothermal flux^49^.

Our recognition of a major geothermal anomaly at the ice divide close to South Pole and within East Antarctica has several significant implications. Firstly, it implies that the preservation of ancient climatic records around the South Pole region is likely not as promising as proposed by several previous continental scale ice sheet models^14^. Inclusion of both our maximum and minimum values of heat flux in glaciological models will allow for a better estimation of the potential spatial distribution of >1 Ma old ice. Secondly, our discovery of an additional water source at the ice divide helps explain the numerous lakes further downstream, including the dynamic systems in the lower parts of the glacial catchments. Such an active hydrological system may in turn influence the onset and maintenance of enhanced ice flow beneath some of Antarctica’s largest glaciers, as suggested for the adjacent Recovery Glacier system^34^. Thirdly, given our interpretation of the origin of the geothermal anomaly we suggest that highly radiogenic rocks and intraplate faulting may exert important influences on basal melting patterns around South Pole. Similar processes may affect other largely unexplored areas of interior East Antarctica. We conclude that higher resolution future geophysical studies and drilling are required to better constrain how geology and geothermal heat flux influences the variability in subglacial and englacial conditions in both East and West Antarctica.

## Methods

To calculate the melt rates and geothermal heat flux responsible for layer drawdown, we analysed the internal layers shown in Fig. 2 following a three step process:

1. Internal layers were dated based on a composite depth-age model constructed using layers in our data linked the South Pole SPICECORE^53^ drill site (Conway and Fudge pers. com. 2017) and layers linked to the Lake Vostok drill site in older reconnaissance radar data^54^ (Supplementary Material 2 and Fig. S2).
2. A 1D depth-age model was used to invert for the basal melt rate and surface accumulation that best fit depth-age patterns in the dated layers along the profile (Fig. 2a) [This included a Monte Carlo approach to propagate errors in both layer age and uncertainties in rheology, (Supplementary Material 1.2)].
3. The geothermal flux was calculated by solving the heat equation^55^, assuming the bed is at the pressure melting point and basal heat-flux balances conduction and downward advection of cold ice from the surface (Supplementary Material 3.2).

All maps were made by the authors for this publication using Geosoft Oasis montaj mapping tools version 8.5, https://www.geosoft.com/. The underlying data for the figures was collected and processed by the authors, or is from publically available sources (see citations).

## Electronic supplementary material

Supplementary material

## Data Availability

The radar data presented in this paper is available from the European Space Agency Earth Observation Campaigns Data web page (https://earth.esa.int/web/guest/campaigns). The presented radargrams are from flights P25, P26 and P31 and are available from the same source.
