# Supplementary material for: Anomalously high geothermal flux near the South Pole
Source: Sci Rep. 2018 Nov 14;8:16785. doi: 10.1038/s41598-018-35182-0 (PMC6235973; doi:10.1038/s41598-018-35182-0)
Supplement: Supplementary file 1 — Supplementary material [file 41598_2018_35182_MOESM1_ESM.pdf]

Anomalously high geothermal flux near the South Pole

Supplementary material

T.A. Jordan*[1], C. Martin[1], F. Ferraccioli[1], K. Matsuoka[2], H. Corr[1], R. Forsberg[3], A. Olesen[3], M. Siegert[4]

1. British Antarctic Survey, High Cross, Madingley Road, Cambridge. CB3 0ET, UK *tomj@bas.ac.uk

2. Norwegian Polar Institute, Tromsø, Norway

3. National Space Institute, Technical University of Denmark, Lyngby, Denmark

4. Grantham Institute and Department of Earth Sciences and Engineering, Imperial College London, South Kensington, London SW7 2AZ, UK

Supplementary figures

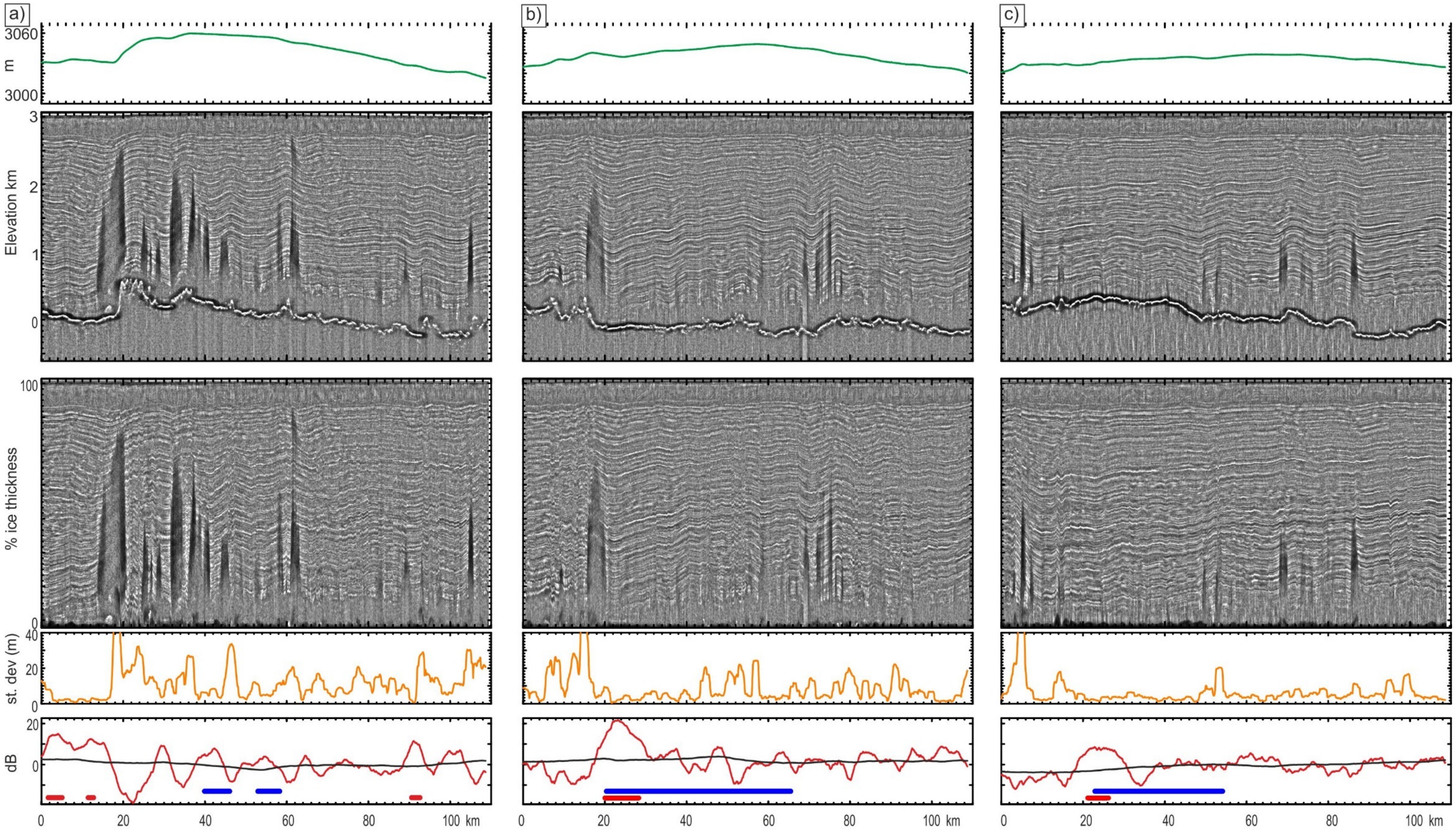

SFig. 1 Radargrams upstream from South Pole. Locations shown in SFig. 2. All profiles oriented with South Pole to the left. Upper panel shows surface elevation. Second panel shows elevation converted radargrams. Note Automatic Gain Correction (AGC) filter applied to enhance layers masks variation in bed brightness. Third panel shows radargrams normalised for ice thickness. Fourth panel shows bed roughness calculated as the standard deviation of residual high frequency topography within a 1.5 km moving window. Fifth panel shows bed return power (prior to AGC filter) corrected for geometric spreading and englacial attenuation. Red line shows local 3 km mean values. Black line shows regional 60 km mean values. Horizontal thick blue and red lines show layer draw down indicative of basal melting and inferred ponded basal water respectively.

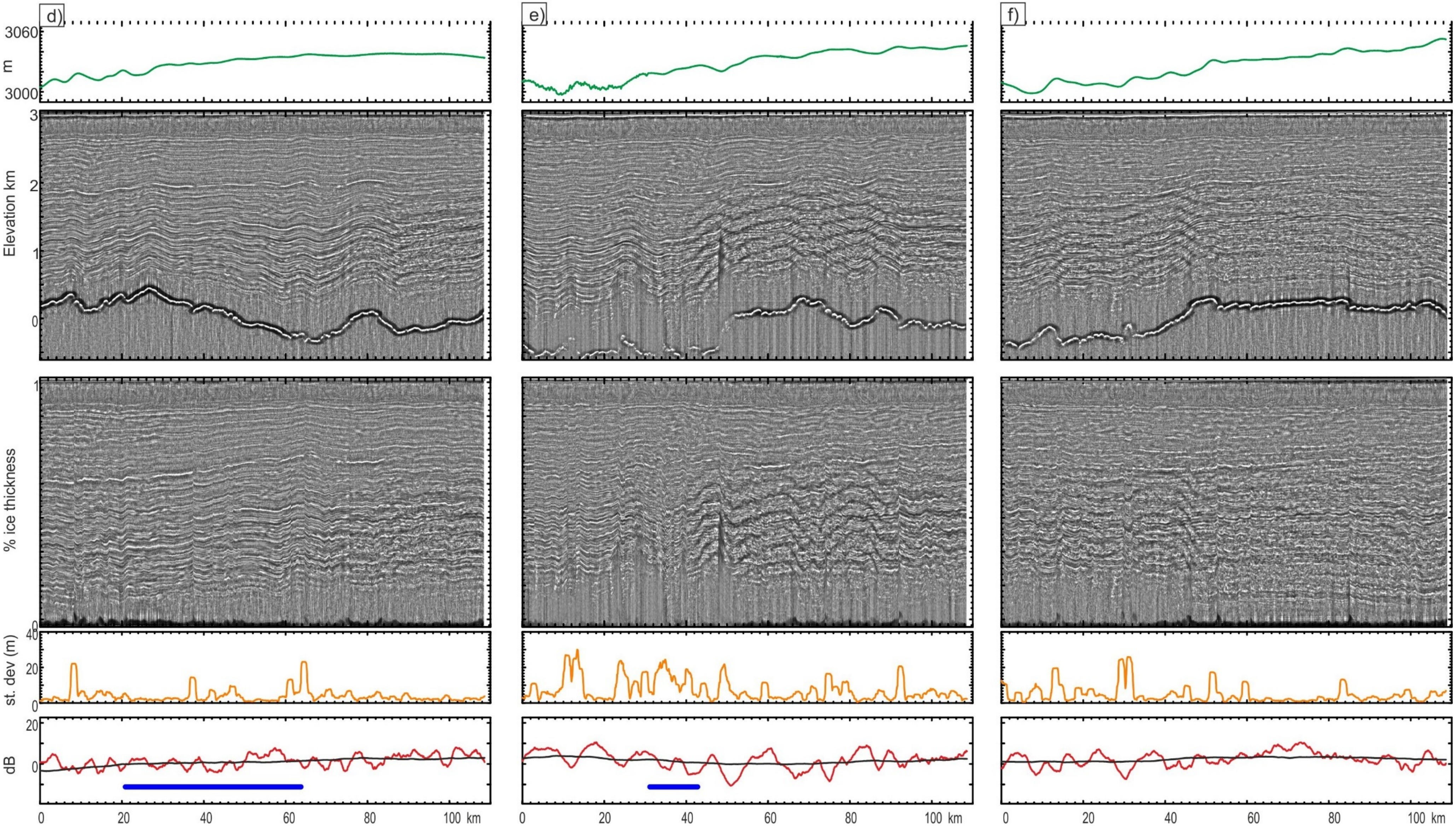

SFig. 1 continued.

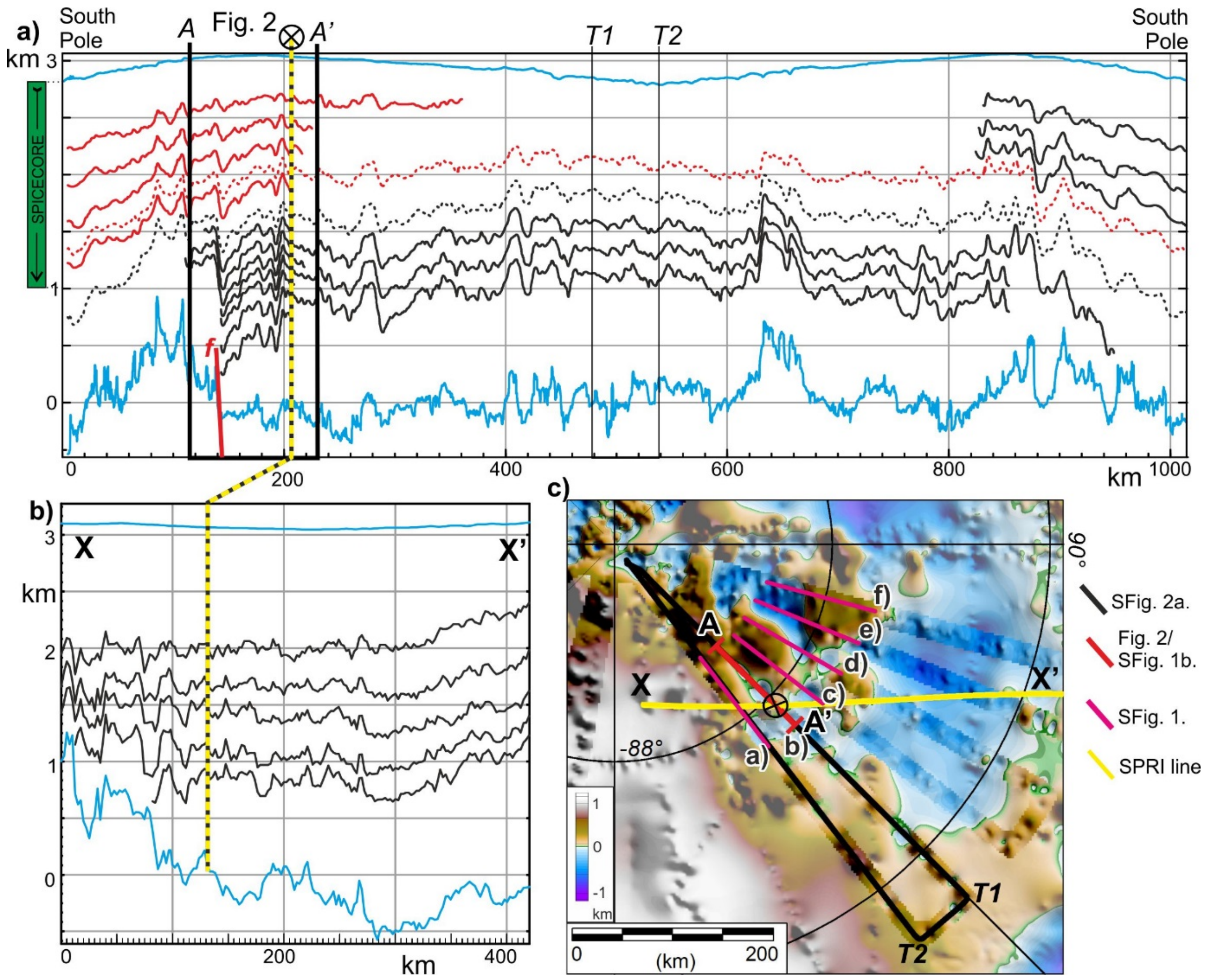

SFig. 2. Traced internal layers. a) Entire PolarGAP flight line including modelled region of enhanced basal melting (main text Fig. 2 A-A'). Blue lines mark picked ice sheet surface and bed. Red layers highlight dated horizons traced from South Pole. T1 and T2 locate turns. Note the deepest layer at Pole is below the currently available SPICECORE depth-age model (green bar). Also note shallow layers are present in the region of T1-T2, but have not been traced for this study. Dotted lines mark horizons traced along the entire flight used to provide an estimate of error in tracing individual layers. Vertical yellow line marks intersection point with SPRI flight. We call this point the "Dipstick" as our initial depth age model was constrained at this location. Red line (*f*) marks proposed fault scarp at margin of subglacial highlands. b) Section of SPRI flight line with dated internal layers traced from the Vostok ice core in black[1]. c) Location map of picked profiles overlain on subglacial topography. Strong colour from PolarGAP survey, light colours show bed elevation interpolated from previous surveys. Short pink and red lines (a-f) locate profiles in SFig. 1. Yellow line marks SPRI profile X-X'. Cross at SPRI/PolarGAP intersection locates the Dipstick.

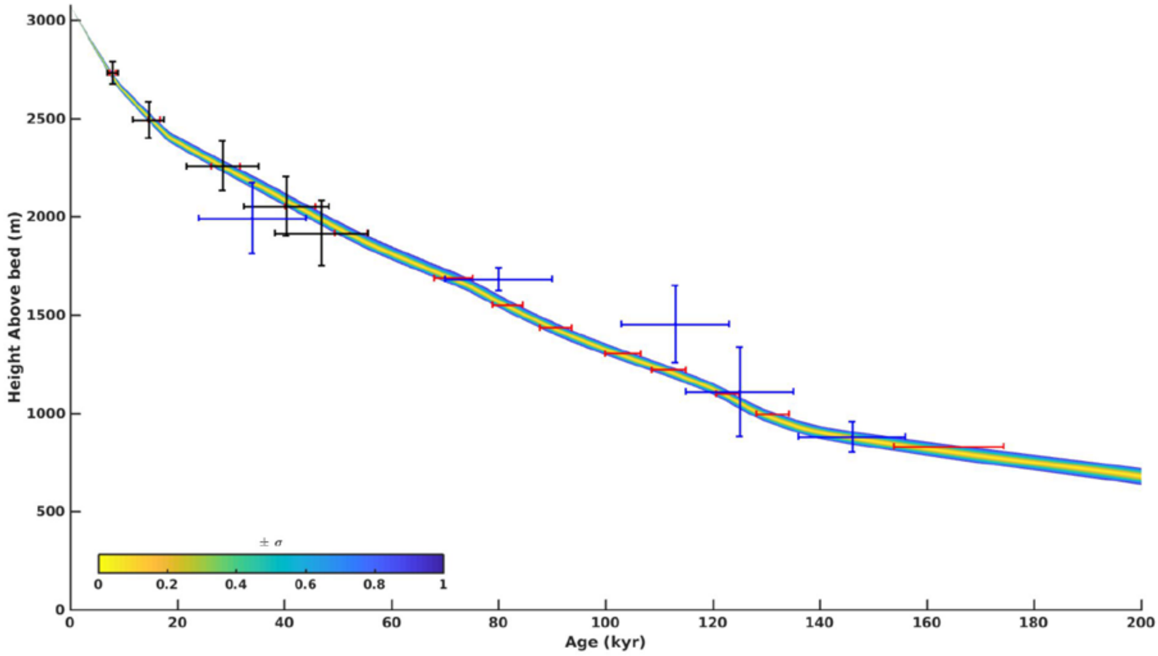

SFig. 3. Depth age curve at PolarGAP/SPRI intersection (Dipstick). Black and blue crosses mark age and uncertainties for layers traced from SPICECORE, and the Vostok Ice core respectively. Depth-age model derived for optimised rheology and recent (1 ka average) accumulation, assuming a fixed zero basal melt rate. Red error bars represent the derived values of age and its uncertainty at the radar layers used in the paper. Note error envelope for depth-age model is narrower than errors on initial data points as knowledge of past temporal variations in accumulation acts as an additional constraint. The +/-1 sigma error bounds in this and subsequent figures were calculated based on the distribution of the results of 1000 runs of our Monte Carlo analysis.

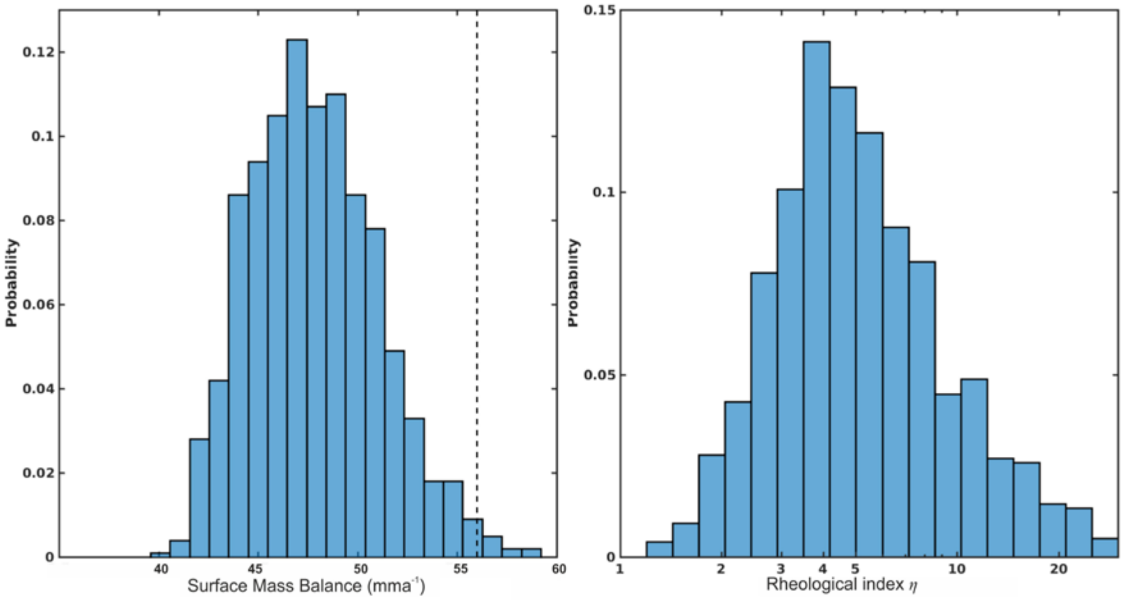

SFig. 4. Propagation of uncertainty at the dipstick for the optimal values of (a) surface mass balance and (b) rheological index η. The dashed line in (a) is the estimated present surface accumulation[2]. The rheological index is the dimensionless flow behaviour index describing the deviation of ice flow response from a linear Newtonian fluid ($\eta = 1$) towards a plastic material ($\eta \gg 1$).

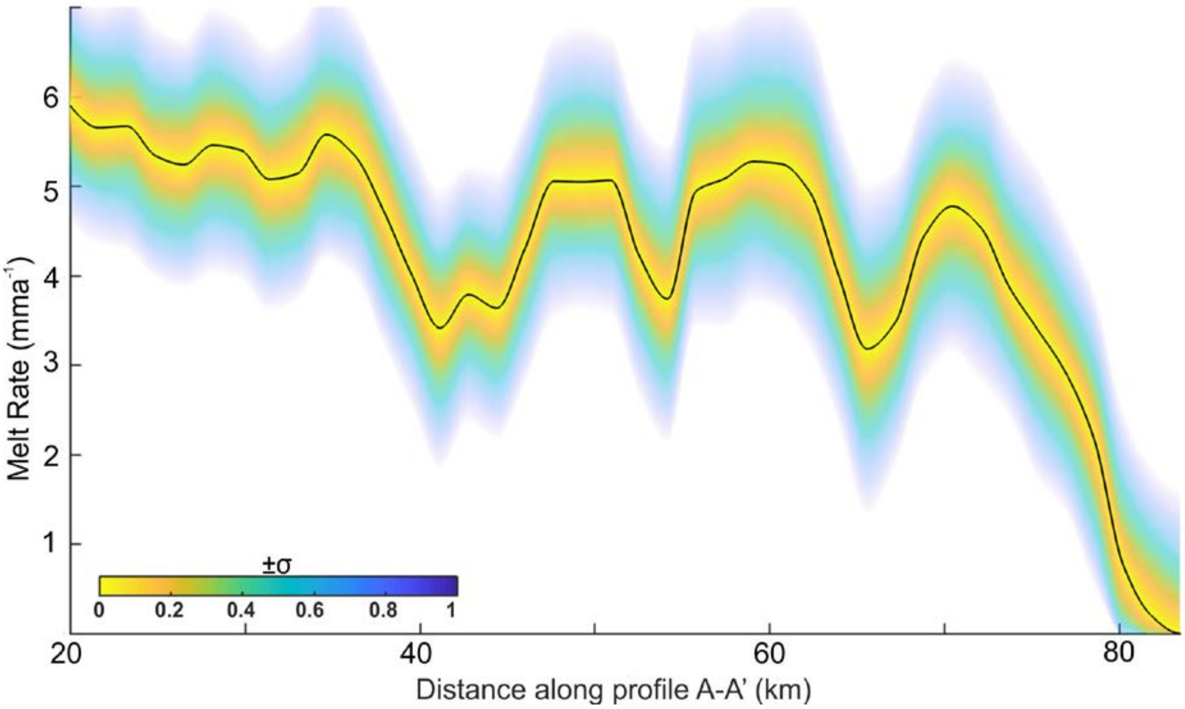

SFig. 5. Calculated basal melt rate along modelled profile. The +/-1 sigma error bounds were calculated based on the distribution of the results of 1000 runs of our Monte Carlo analysis.

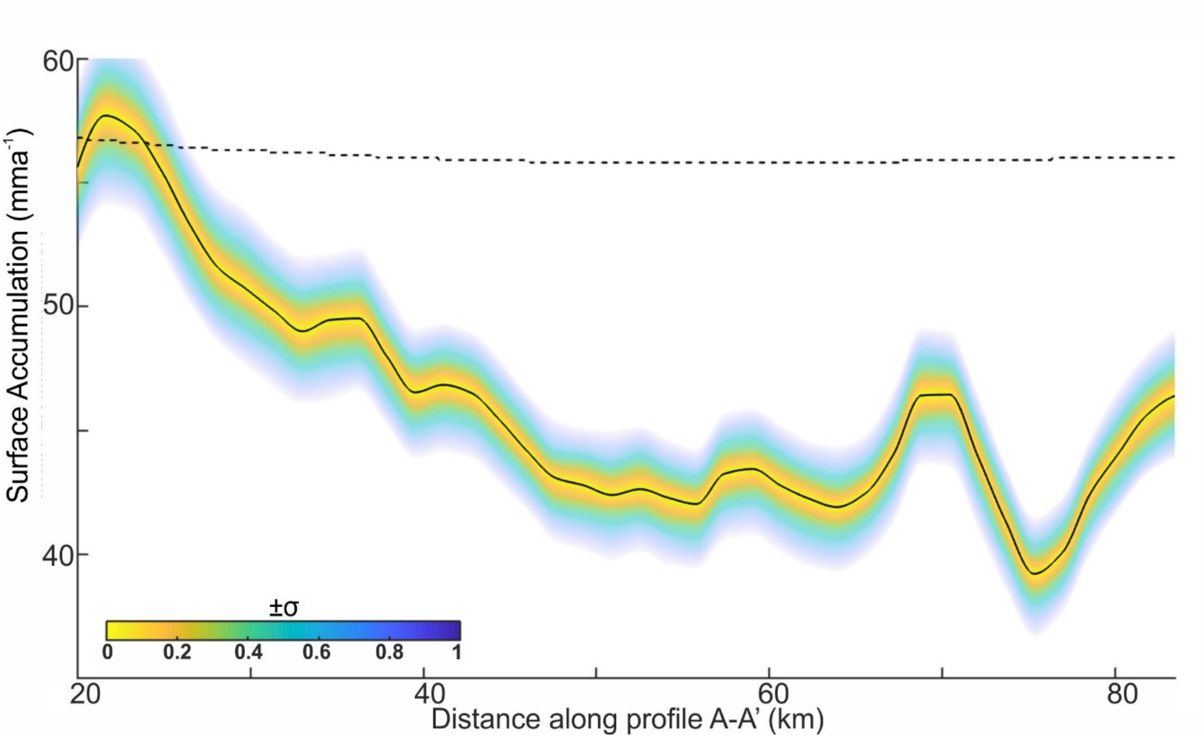

SFig. 6. Recovered spatial variation in accumulation rate along modelled profile. Dashed line marks regional estimate of present day accumulation rate[2]. An average accumulation value for the last few thousand years ~15% lower than present day accumulation is consistent with observations elsewhere in Antarctica[3].

Supplementary Table 1

Layer Depth 22 km from Pole	Layer Depth at SPRI intersection (Dipstick)	Age yr	Depth error m	Age error yr	Survey/ age source
603	372	7944	57	941	PolarGAP/SPICECORE
931	612	14602	92	2859	PolarGAP/SPICECORE
1244	846	28488	125	6685	PolarGAP/SPICECORE
1486	1052	40371	151	7959	PolarGAP/SPICECORE
1613	1187	46955	165	8743	PolarGAP/SPICECORE
	1112	34000		10000	SPRI/Vostok
	1421	80000		10000	SPRI/Vostok
	1651	113000		10000	SPRI/Vostok
	1995	125000		10000	SPRI/Vostok
	2224	146000		10000	SPRI/Vostok

Supplementary Table 1: Input depth-age relationship and errors for layers at the PolarGAP/SPRI intersection point (Dipstick) (See SFig. 3). Errors in depth and age are derived from uncertainties in layer tracing. Uncertainties in age/depth from ice core records are assumed to be negligible in comparison.

Supplementary text

1. Data collection and processing

1.1. Radar data and bed elevation

The geophysical observations presented in this paper were made as part of the European Space Agency (ESA) PolarGAP airborne survey (Fig. 1), which collected ~30,000 km of line-track data during the 2015/16 field season. Radargrams and ice thickness estimates were made with the British Antarctic Survey coherent radar system (PASIN), which has a carrier frequency of 150 MHz, a bandwidth of 12 MHz and acquires pulse-coded waveforms at a rate of 312.5 Hz. To reduce the off-axis scattering and improve the signal to noise a Doppler beam sharpening filter was applied to the data. Then to identify the bed echo the data was first decimated to 2 Hz, giving an along track spacing of ~30 m. The onset of the bed echo was identified and picked in a semi-automatic manner using PROMAX seismic processing software. The travel time between the surface and the bed pick was converted to ice thickness using an EM velocity in ice of 168 mµ s^{-1}, a standard firn correction of 10 metres was then added. Crossover analysis comparing recovered bed elevation (surface elevation minus ice thickness) at line intersections across the entire survey area yielded an overall RMS error of 65.7 m. This number includes a small number of large errors >200 m seen in mountainous areas, reflecting places where the bed is picked at different elevations on different lines – likely reflecting misidentification of off axis reflections as bed returns. For regions with less extreme topography, as seen upstream of South Pole, the RMS error estimate for the PolarGAP survey from cross over analysis is 31 m.

1.2. Basal reflectivity

To examine the spatial variation in basal reflectivity R, we assume the bed is specular and recover the relative Fresnel reflection coefficient using a simplified form of the radar equation. The maximum amplitude A within a 100 ns window below the picked bed return was extracted from the radar data. The recovered echo power was corrected for geometric spreading and attenuation in the ice (K) as follows:

$$[R] = 20 \times log10\, A + Gcor + (2 \times K \times tice) + (Offset) \quad (1)$$

Where $tice$ = ice thickness and $Gcor$ is the geometric correction calculated as:

$$Gcor = 20 \times log10\, ((E_a - E_s) + (tice/\varepsilon^{0.5})) \quad (2)$$

Where E_a = aircraft elevation, E_s = ice surface elevation and permittivity of ice $\varepsilon = 3.2$, the *[]* donate a dB scale. As the dB scale is relative we shifted data on all profiles by -135 dB ($Offset$) which gave a mean value of zero across the three flights flown upstream of South Pole.

The term due to attenuation within the ice column assumes a fixed attenuation rate of 10 $dBkm^{-1}$ (one way), consistent with previous estimates for the interior of the East Antarctic Ice sheet[4]. The ice thickness along the model profile varies between 2711 and 3293 m, and hence the variation in the geometric correction along the profile is <1.5 dB. We assume that attenuation rate is uniform along our comparatively short profile. Attenuation rate may vary with ice sheet temperature in response to variability in accumulation rate and geothermal flux[5]. However, the limited variation in modelled accumulation rate from ~40 to 58 mm a^{-1} implies an associated change in attenuation of just ~1 dB km^{-1}, or ~6 dB in total[5]. The observed variability in reflectivity is >20 dB indicating we are observing basal properties rather than variability ice sheet attenuation. In addition the basal brightness shows an abrupt step in power, which is not predicted by models of the accumulation or ice sheet thermal structure.

Areas with 'bright' bed were defined as those regions where the local reflectivity, after a 3 km mean filter, was 10 dB greater than the regional reflectivity, defined by a 60 km filter of the same data. This comparison further helps to remove the impact of any regional variability in attenuation. The brightness threshold was chosen as +10 dB power has been used as an indicator of subglacial water by previous workers[6].

Bed reflectivity is enhanced by both the presence of water and the smoothness of the bed[5]. We therefore calculated a measure of bed roughness and compare it with the recovered basal reflectivity (SFig. 1). The standard deviation within a moving window gives a simple estimate of the of the roughness of a surface[7]. To minimise the impact of regional slopes the bed elevation was high-pass filtered before the standard deviation was calculated. We chose an analysis window of 1.5 km for the high pass filter and for assessment of the standard deviation. This width is narrower than the main topographic features, and hence gives a view of relatively local bed roughness. Such a statistical measurement of basal roughness is not a direct measure of the meter-scale roughness, which causes scattering and attenuation of the radar signal. However, studies have shown that qualitatively statistical and direct radar measurements of bed roughness appear to reveal the same patterns[8], hence it is reasonable to use the statistical measure as a proxy for the expected variation in attenuation due to roughness. More complete analysis of the self-affine nature of subglacial roughness are beyond the scope of this paper. Our results (SFig. 1) show that there is not a direct correlation between smooth and bright bed, indicating that the presence of water, rather than variation in roughness alone, is the most likely contributor to the observed reflectivity signal.

1.3. Ice sheet surface elevation

A Riegl Q-240i scanning lidar was used to measure the surface elevation for the majority of PolarGAP flights, including along the analysed flight profiles. Data were corrected for roll, pitch and heading from an on board iMAR high-accuracy inertial measurement unit, calibrated from known target over-flights and Rothera and South Pole research stations, and combined with a high precision kinematic dual-frequency GPS position solution. Lidar surface elevation estimates are considered to be accurate to better than 10 cm, as confirmed by the swath cross-overs throughout the PolarGap survey. To simplify further analysis, the surface elevation directly below the aircraft at each radar sampling point was interpolated from the lidar swath. For some of the PolarGAP flights lidar data was unavailable, or intermittent, due to low clouds or fog/snowdrift. In these cases the ice surface was derived from the ice surface return picked by the radio-echo sounding system and calibrated to regions where lidar data were available. In areas where radar data was used the surface elevation is accurate to ~1.2 m.

2. Depth Age model

To estimate basal melting we utilised a series of dated ice sheet internal layers (assumed to be isochronous) which extend across the region of proposed melting. These dated layers were used to constrain a transient 1D depth-age model, allowing estimation of optimal basal melt and surface accumulation rates. The procedures for identifying layers, estimating errors and details of the model used are laid out below.

2.1. Tracing internal layers.

Internal layers (SFig. 2) were manually picked from the radargrams as follows. Radargrams were first transformed to rasters, where the x axis is trace number and the y axis is elevation calculated assuming a radar velocity in ice of 168 m μs^{-1}. A Gaussian filter with a 10 trace wide (~300 m) and 10 m deep window was applied to the raster to minimise random noise in the radar data. Layers were enhanced by either an automatic gain correction (AGC) filter, or by calculating the derivative of the

radar amplitude with depth. Layers were then manually digitised, primarily by following the edges of high amplitude layers in the AGC data, but also guided by derivative information in regions where layers were difficult to follow in the AGC data. The digitised information (trace number and elevation) for each layer was then re-combined with spatial navigation information for display and further modelling.

2.2. *Initial depth-age values and associated uncertainty*

The shallowest five layers traced from close to South Pole (SFig. 2a) were initially assigned ages based on the depth-age curve (Conway and Fudge (pers. com. 2017)) derived from the SPICECORE[9] drill hole, assuming radar layers in the vicinity of South Pole are horizontal. To assess the uncertainties associated with layer tracing two layers with initial elevations of 746 m and 1351 m were traced for ~1000 km forming a closed loop (SFig. 2a). Discrepancies in elevation at the loop closure were 17 and 58 m for the shallow and deep layers respectively. This suggests an average error in traced layer depth of ~37 m. An additional uncertainty in layer age stems from the assumption of horizontal layers upstream of South Pole. The model line starts ~22 km from the SPICECORE drill site introducing significant potential errors. An adjacent line extending to within 4 km of the SPICECORE site suggests that over 22 km upstream of South Pole layers ~600 m deep vary by just 21 m, while layers ~1600 m deep vary by up to 130 m. The overall error in depth was assumed to reflect the sum of the average uncertainty in traced layer depth, and an error proportional to layer depth due to the assumption of horizontal layers upstream of South Pole. The associated uncertainty in age was calculated by applying the maximum depth uncertainty to the SPICECORE depth-age model (Conway and Fudge (pers. com. 2017)). To provide age information for the deeper parts of the profile we used data from the intersection with a radar line flown during the SPRI campaign where layers originating close to the Vostok ice core have been traced[1] (SFig. 2b). The uncertainty in age for the layers traced from Vostock in the SPRI data was assumed to be 10 ka. Input data for the initial depth-age model is shown in Supplementary Table 1.

2.3. *Dating the dipstick*

Although the five shallowest layers can be dated to an age of ~47 ka by association with the SPICECORE drill site, the reminder of layers were too deep (SFig. 2a). The intersection between the PolarGAP line and an older SPRI flight, known as the Dipstick, meant we were able to augment our depth-age information with layers traced from the Vostock ice core[1] with dates stretching back to >140 ka. However, as it can be challenging to directly link specific dated radar layers imaged by different radar systems[10] we instead constructed a depth age model constrained by both data sets (SFig. 3), and assigned intersecting layers ages from this model. The best fitting depth-age mode was constructed using the numerical model described below in Section 2.4. This technique was used in favour of simply extrapolating information between dated layers as age-depth is highly non-linear. Ages and uncertainties for all layers intersecting the dipstick, including those previously dated by proximity with SPICECORE, were derived from the best fitting model to ensure consistency in error propagation with depth.

We assume that there is no melting beneath the dipstick location, as there is no evidence of layer drawdown or a bright basal reflector in this location (Main text Fig. 2, SFig. 1b). We estimate the optimal values of the rheological index n and present surface accumulation so that the difference in age between model and observations traced from the ice-cores is fit best. The rheological index η is the dimensionless flow behaviour index, which describes the deviation of ice flow response from a linear Newtonian fluid ($\eta = 1$) towards a plastic material ($\eta \gg 1$). For the minimization, we use the Nelder-Mead simplex algorithm[11] within Matlab R2017a (fminsearch). We use a basic Monte Carlo method[12] to propagate the uncertainty in dates traced from the ice-cores to the age-depth model

used for dating the radar layers. The propagation of uncertainty is obtained as the distribution of probability from a set of one thousand results for the optimal rheological index η, surface accumulation and the derived age-depth model. For each one of the simulations, we resample the measured age-depth observations assuming a uniform distribution within the area of confidence of the observations. The recovered optimal values for η and present (~1 ka average) accumulation, and their uncertainty, are shown in SFig. 4. These values are reasonable given previous studies of recent (~1 ka average) Antarctic accumulation[3] and ice sheet rheology, which has an expected range of between 2 and 5[13,14].

Some models by other authors[15] suggest that there may be regionally widespread melting in the area of the Dipstick of around 3 mm a^{-1}. Such regional melting would not be resolved as local layer drawdown and could theoretically affect our recovery of a reliable depth age curve. To test if our assumption of zero melting at the Dipstick was valid we therefore re-ran the inversion imposing a melt rate of 3 mm a^{-1}. This test recovered values for η which were highly skewed to values of one (the minimum allowed in our inversion). Recovery of such skewed and unreasonably low values[13] for η indicate that at this specific location significant basal melting is not consistent with the depth age curve indicated by tracing internal layers (SFig. 3). Hence, our assumption of zero melting at the dipstick is likely valid.

2.4. The transient 1d depth-age model

For this paper, we consider that the distribution of the age varies in time, from a given initial state $\boldsymbol{A_0}$ to the present ($t = t_p$), but we neglect the effect of horizontal advection and the local variation of thickness through time ($\frac{\partial H}{\partial t}$= 0). The age of ice can then be expressed as:

$$\begin{aligned} \partial_t A + w(x,y,z,t)\partial_z A = 1, \quad 0 \le z \le H, 0 \le t \le t_p, \\ A(x,y,z,0) = A_0, \quad 0 \le z \le H, \\ A(x,y,H,t) = 0, \quad 0 \le t \le t_p. \end{aligned} \tag{3}$$

We assume that the vertical velocity takes the form[16]:

$$w(x,y,z,t) = -m(x,y) + [-a(x,y,t) + m(x,y)]\,\eta(z). \tag{4}$$

where a is the surface accumulation rate, m the basal melt rate, and η is the shape function that only depends on rheology and not on time dependent variables such as surface accumulation or ice thickness. This assumption is supported by perturbation analysis for isothermal ice[17]. For the sake of simplicity, we refer to the surface accumulation rate and basal melt rate as accumulation and melting, as there is no possible ambiguity in this paper. As thickness H is assumed to be constant through time unaccounted for variations in thickness will be reflected in our inversion results as variations in the recovered accumulation rate, as both factors have approximately the same impact on the depth of the internal layers.

We further assume that the temporal variation of accumulation is proportional to that at Dome C (a_{DC}) for the last 800,000 years[18] and that melting, at a given location, does not vary through time,

$$a(x,y,t) = a(x,y,t_p)\frac{a_{DC}(t)}{a_{DC}(t_p)} \tag{5}$$

We also assume that the shape function η follows a shallow ice approximation[19]:

$$\eta(s) = 1 - s\left(\frac{n+2}{n+1} - \frac{s^{n+1}}{n+1}\right) \tag{6}$$

where s is the normalized depth ($s = (H - z)/H$).

Regarding the initial condition for Equation (3), A_0, it has no influence on the age solution because the simulation time t_p is 800,000 years, older than the deepest layer in our study estimated with any combination of realistic parameters.

To summarize, for a given surface accumulation, rheological index and melting, the vertical velocity is defined (Eq. (4)) and Equation (3) is a closed problem. Numerically, we solve Equation (3) using a two time levels Semi-Lagrangian algorithm[20].

3. Melt rate and geothermal heat flux determination

3.1. Basal melt calculation

Once the full set of radar layers were dated at the dipstick, we use them to estimate the vertical velocity at each point along the profile using the numerical model described in Section 2.4. In our model, vertical velocity is a combination of surface accumulation, rheology and basal melting; these are parameterized by the present (~1 ka) accumulation $a(x, y, t_p)$, rheological index $\eta(x, y)$ and basal melt rates $m(x, y)$.

We find that the age-depth relationship is highly sensitive to both rheology and melting near the bottom where the model is least constrained by dated layers (there is no dated radar layers in the deepest third of the ice thickness). The consequence is that we cannot simultaneously estimate basal melting and rheology with our dataset. Instead, we assume that the rheology can only vary spatially within the range and the distribution that we estimated at the dipstick (SFig. 4b), where rheology was estimated in the assumed absence of melting.

On the contrary, near the surface our age-depth model is most sensitive to the values of recent (~1 ka) surface accumulation and nearly independent of the values of the rheological index or basal melting. Also, the top half of the ice thickness is populated by radar layers well constrained by links to the SPICECORE drill site and hence provides good constraint for the age-depth model.

To propagate the value and uncertainty in age-depth and rheology at the dipstick into our estimates for basal melting and present surface accumulation along the profile, we use a basic Monte Carlo method[12], as in Section 2.3. The error propagation is estimated by the probability distribution of a set of one thousand results for the optimal surface accumulation and basal melt rates and the derived age-depth. For each one of the simulations, we sample the age of the radar layers and the rheological index η from the distribution of optimal values obtained at the dipstick. The optimal surface accumulation and basal melting are the ones that minimize the difference in age between model and dated layers. As in the previous Section, we use the Nelder-Mead simplex algorithm[11] within Matlab R2017a (fminsearch) to recover the optimal surface accumulation and basal melting values for each run.

The estimated melting rates are in SFig. 5 and the recovered variation in surface accumulation is in SFig. 6.

3.2. *Estimating heat flux from basal melt rates*

The heat transfer at the ice-bed interface can be summarized as:

$$Q_S + Q_w + Q_L + Q_c + Q_G = 0\,, \tag{7}$$

where Q_S is the heat generated by sliding, Q_w is the heat generated by subglacial water flow,Q_L is the latent heat used by melting, Q_c is the heat conducted by the ice to the surface and, finally, Q_G is the geothermal heat flux that is the variable we want to estimate[13].

Our area of study is in an area with low horizontal surface velocity[21], and calculations based on the work of Arthern et al 2014[22] suggest Drag Heating of just 0.098 mWm^{-2} in this area. It is therefore reasonable to assume that geothermal heat flux is the dominant driver for melting in this region and hence we neglect Q_S. Also the subglacial topography shows that the area is at the top of the hydraulic catchment area and we do not expect the presence of significant subglacial water flux so we neglect the effect of subglacial water flow Q_w.

The latent heat can be derived from the estimated melting rates and the conducted heat can be derived from the vertical gradient of temperature in the areas where we estimate positive melting. In this area, we can assume that the basal temperature is at the basal pressure melting point T_m, as layer draw-down demonstrates that melting is occurring. We therefore derive T_m from the hydrostatic pressure as being proportional to ice thickness[23], and we solve the heat equation from an initial temperature T_0 to the present solution at time t_p,

$$\partial_t T + w(x,y,z)\partial_z T - \partial_z(k\partial_z T) = 0, \tag{8a}$$

$$T(z,0) = T_0(z), \tag{8b}$$

$$T(H,t) = T_s(x,y,t), \tag{8c}$$

$$T(0,t) = T_m \tag{8d}$$

where k is the thermal diffusivity, the vertical velocity vary spatially and temporally as estimated in the previous section. We neglect the effect of horizontal advection or conduction and assume that the surface temperature is proportional to the measured present values[24] and temporal variations follow the values estimated at Dome C over the last 800,000 years[18],

$$T_s(x,y,t) = T_s(x,y,t_p)\frac{T_{DC}(t)}{T_{DC}(t_p)} \tag{9}$$

As the simulation over 800,000 years is larger than the conduction and advection time-scales, the effect of the initial condition is negligible, we solve Equation (8) using a Crank-Nicolson scheme[25].

We then estimate our values for the Geothermal heat flux as,

$$Q_G(x,y) = -\rho_w L m(x,y) - k\frac{\partial T(x,y,z,t_p)}{\partial z}|_{z=0} \tag{10}$$

As in the previous steps, we propagate the uncertainty to our estimates with a basic Monte Carlo method. We sample the basal melting, surface accumulation and rheological index at each location

along the profile as derived in the previous section, and solve for the geothermal heat flux by solving Equations (8)- (10). Our resulting estimates are in the main text Fig. 2c.

It is important to note that we have assumed that the basal temperature equals the pressure melting point. This is reasonable given the layer drawdown indicates basal melting, and hence the basal temperature must be at the pressure melting point. In an area near the dipstick, highlighted with a blue box in Fig. 2c, this is not true according to our estimates of zero melting. In that area, our estimate is the maximum value of the geothermal flux.

4. Basal hydrology

To calculate basal hydrology our new surface and bed elevation information was integrated with existing topographic data. Point bed elevation values along flight lines from the PolarGAP, AGAP[26] and SPRI/NSF/TUD surveys[27] were merged into a single database. In addition gridded bed elevation information from the Pensacola Pole traverse survey[28] were re-sampled onto the location of their original flight lines and merged with the available survey line data. The line bed elevation information was then gridded using a minimum curvature approach with a cell size of 2.5 km.

Surface elevation outside 88°S was derived from the CRYOSAT 2 digital elevation model (DEM)[29]. South of 88°S our new lidar data reveals errors of ~150 m in the CRYOSAT 2[29] and BEDMAP2[30] surface DEM due to the lack of satellite data coverage. From a hydrological perspective such discrepancies equate to >1500 m errors in bed elevation. We therefore developed an improved preliminary surface DEM based on our new along track Lidar data. To generate this more accurate DEM within the Polar gap we converted the CRYOSAT 2 DEM to point values and masked all values south of 88°S. Along track estimates of surface elevation from the PolarGAP and AGAP surveys were then added to the database and the surface elevation re-gridded onto a 2.5 km mesh using a minimum curvature algorithm. Although line spacing is up to 30 km south of 88°S the low surface slopes, often <1 m km^{-1}, mean that the resulting estimates of surface elevation are likely to be reasonable, and represent a significant improvement on previous estimates.

To calculate hydrology and basal water routing we followed a four part approach implemented in ArcGIS. First the hydrological potential[31] (Hp) assumed to drive subglacial water flow was calculated from our new surface (E_s) and bed (E_b) DEM as:

$$Hp = (g \times \rho w \times E_s) + (g \times \rho i \times (E_s - E_b))$$

Where g is the normal gravity, ρw and ρi represent density of water and ice respectively. Secondly all local hydrological minima (sinks) were filled to give continuous flow to the edge of the model. Thirdly the direction of flow was calculated from the filled hydrological potential model[32]. Finally flow accumulation across the hydrological model was calculated[33]. Flow accumulation gives a count of the number of upstream cells feeding any given point, revealing the main subglacial drainage networks (main text Fig. 3).

References.

1 Siegert, M. J. Glacial–interglacial variations in central East Antarctic ice accumulation rates. *Quaternary Science Reviews* **22**, 741-750, doi:http://dx.doi.org/10.1016/S0277-3791(02)00191-9 (2003).

2 Arthern, R., Winebrenner, D. P. & Vaughan, D. G. Antarctic snow accumulation mapped using polarization of 4.3-cm wavelength microwave emission. *Journal of Geophysical Research* **111**, doi:10.1029/2004JD005667 (2006).

3 Fujita, S. *et al.* Spatial and temporal variability of snow accumulation rate on the East Antarctic ice divide between Dome Fuji and EPICA DML. *The Cryosphere* **5**, 1057-1081, doi:10.5194/tc-5-1057-2011 (2011).

4 Matsuoka, K., MacGregor, J. A. & Pattyn, F. Predicting radar attenuation within the Antarctic ice sheet. *Earth and Planetary Science Letters* **359**, 173-183, doi:http://dx.doi.org/10.1016/j.epsl.2012.10.018 (2012).

5 Matsuoka, K. Pitfalls in radar diagnosis of ice-sheet bed conditions: Lessons from englacial attenuation models. *Geophysical Research Letters* **38**, doi:10.1029/2010GL046205 (2011).

6 Fujita, S. *et al.* Radar diagnosis of the subglacial conditions in Dronning Maud Land, East Antarctica. *The Cryosphere* **6**, 1203-1219, doi:10.5194/tc-6-1203-2012 (2012).

7 Jordan, T. A. *et al.* Hypothesis for mega-outburst flooding from a palaeo-subglacial lake beneath the East Antarctic Ice Sheet. *Terra Nova* **22**, 283-289, DOI: 210.1111/j.1365-3121.2010.00944.x (2010).

8 Jordan, T. M. *et al.* Self-affine subglacial roughness: consequences for radar scattering and basal water discrimination in northern Greenland. *The Cryosphere* **11**, 1247-1264, doi:10.5194/tc-11-1247-2017 (2017).

9 Casey, K. A. *et al.* The 1500m South Pole ice core: recovering a 40ka environmental record. *Ann. Glaciol.* **55**, 137-146, doi: 110.3189/2014AoG3168A3016 (2014).

10 Winter, A. *et al.* Comparison of measurements from different radio-echo sounding systems and synchronization with the ice core at Dome C, Antarctica. *The Cryosphere* **11**, 653-668, doi:10.5194/tc-11-653-2017 (2017).

11 Lagarias, J. C., Reeds, J. A., Wright, M. H. & Wright, P. E. Convergence Properties of the Nelder--Mead Simplex Method in Low Dimensions. *SIAM Journal on Optimization* **9**, 112-147, doi:10.1137/s1052623496303470 (1998).

12 Campolongo, F., Saltelli, A., Sorensen, T. & Tarantola, S. in *Sensitivity Analysis* (eds A. Saltelli, K. Chan, & M. Scott) 15-47 (Wiley, 2000).

13 Cuffey K.M. & W.S.B., P. *The physics of glaciers*. 4th edn, (Elsevier, 2010).

14 Gillet-Chaulet, F., Hindmarsh, R. C. A., Corr, H. F. J., King, E. C. & Jenkins, A. In-situquantification of ice rheology and direct measurement of the Raymond Effect at Summit, Greenland using a phase-sensitive radar. *Geophysical Research Letters* **38**, n/a-n/a, doi:10.1029/2011GL049843 (2011).

15 Pattyn, F. Antarctic subglacial conditions inferred from a hybrid ice sheet/ice stream model. *Earth and Planetary Science Letters* **295**, 451-461, doi:http://dx.doi.org/10.1016/j.epsl.2010.04.025 (2010).

16 Parrenin, F. *et al.* 1-D-ice flow modelling at EPICA Dome C and Dome Fuji, East Antarctica. *Clim. Past* **3**, 243-259, doi:10.5194/cp-3-243-2007 (2007).

17 Wilchinsky, A. V. & Chugunov, V. A. Modelling ice-divide dynamics by perturbation methods. *J. Glaciology* **43**, 352-358, doi:10.1017/S0022143000003300 (2017).

18 Parrenin, F. *et al.* The EDC3 chronology for the EPICA Dome C ice core. *Clim. Past* **3**, 485-497, doi:10.5194/cp-3-485-2007 (2007).

19 Hutter, K. *Theoretical Glaciology.*, (Dordrecht, 1983).

20 Staniforth, A. & Côté, J. Semi-Lagrangian Integration Schemes for Atmospheric Models- A Review. *Monthly Weather Review,* **119**, 2206-2223 (1991).

21 Rignot, E. J., Mouginot, J. & Scheuchl, B. Ice Flow of the Antarctic Ice Sheet. *Science* **333**, 1427-1430. doi 1410.1126/science.1208336. (2011).

22 Arthern, R. J., Hindmarsh, R. C. A. & Williams, C. R. Flow speed within the Antarctic ice sheet and its controls inferred from satellite observations. *Journal of Geophysical Research: Earth Surface* **120**, 1171-1188, doi:10.1002/2014JF003239 (2015).

23 Paterson, W. S. B. *The physics of glaciers*. 3 rd edn, (Pergamon/Elsevier Science 1994).

24 Comiso, J. C. Variability and Trends in Antarctic Surface Temperatures from In Situ and Satellite Infrared Measurements. *Journal of Climate* **13**, 1674-1696, doi:10.1175/1520-0442(2000)013<1674:vatias>2.0.co;2 (2000).
25 Crank, J. & Nicolson, P. A practical method for numerical evaluation of solutions of partial differential equations of the heat-conduction type. *Mathematical Proceedings of the Cambridge Philosophical Society* **43**, 50-67, doi:10.1017/S0305004100023197 (2008).
26 Bell, R. E. *et al.* Widespread Persistent Thickening of the East Antarctic Ice Sheet by Freezing from the Base. *Science* **331**, 1592-1595, doi: 1510.1126/science.1200109 (2011).
27 Drewry, D. J. (University of Cambridge. Scott Polar Research Institute., Cambridge, 1983).
28 Davis, M. B. *Subglacial Morphology and Structural Geology in the Southern Transantarctic Mountains from Airborne Geophysics* M.S. Thesis thesis, Univ. of Texas, (2001).
29 Helm, V., Humbert, A. & Miller, H. Elevation and elevation change of Greenland and Antarctica derived from CryoSat-2. *The Cryosphere* **8**, 1539-1559, doi:1510.5194/tc-1538-1539-2014 (2014).
30 Fretwell, P. *et al.* Bedmap2: improved ice bed, surface and thickness datasets for Antarctica. *The Cryosphere* **7** (2013).
31 Shreve, R. L. Movement of water in glaciers. *J. Glaciology* **11**, 205-213 (1972).
32 Tarboton, D. G. A new method for the determination of flow directions and upslope areas in grid digital elevation models. *Water Resources Research* **33**, 309-319, doi:10.1029/96WR03137 (1997).
33 Tarboton, D. G., Bras, R. L. & Rodriguez-Iturbe, I. On the extraction of channel networks from digital elevation data. *Hydrological Processes* **5**, 81-100, doi:10.1002/hyp.3360050107 (1991).
